# Shock induced endotheliopathy (SHINE) in acute critical illness - a unifying pathophysiologic mechanism

**DOI:** 10.1186/s13054-017-1605-5

**Published:** 2017-02-09

**Authors:** PärIngemar Johansson, Jakob Stensballe, SisseRye Ostrowski

**Affiliations:** 1 Capital Region Blood Bank, Rigshospitalet Section for Transfusion Medicine, Rigshospitalet, Copenhagen University Hospital, Blegdamsvej, 9DK-2100 Copenhagen, Denmark; 2 Department of Surgery, University of Texas Health Medical School, Houston, TX USA; 3 Centre for Systems Biology, The School of Engineering and Natural Sciences, University of Iceland, Reykjavik, Iceland; 4 Department of Anesthesia, Centre of Head and Orthopedics, Rigshospitalet, Copenhagen University Hospital, Copenhagen, Denmark

## Abstract

One quarter of patients suffering from acute critical illness such as severe trauma, sepsis, myocardial infarction (MI) or post cardiac arrest syndrome (PCAS) develop severe hemostatic aberrations and coagulopathy, which are associated with excess mortality. Despite the different types of injurious “hit”, acutely critically ill patients share several phenotypic features that may be driven by the shock. This response, mounted by the body to various life-threatening conditions, is relatively homogenous and most likely evolutionarily adapted. We propose that shock-induced sympatho-adrenal hyperactivation is a critical driver of endothelial cell and glycocalyx damage (endotheliopathy) in acute critical illness, with the overall aim of ensuring organ perfusion through an injured microvasculature. We have investigated more than 3000 patients suffering from different types of acute critical illness (severe trauma, sepsis, MI and PCAS) and have found a potential unifying pathologic link between sympatho-adrenal hyperactivation, endotheliopathy, and poor outcome. We entitled this proposed disease entity, shock-induced endotheliopathy (SHINE). Here we review the literature and discuss the pathophysiology of SHINE.

## Background

Acute critical illness such as trauma, sepsis, myocardial infarction (MI) and post cardiac arrest syndrome (PCAS) affects more than five million patients in the EU annually [1]. Approximately one quarter of acutely critically ill patients develop severe hemostatic aberrations resulting in coagulopathy [2–4], which in patients suffering from severe injury is entitled trauma-induced coagulopathy (TIC) [4, 5], and in patients with sepsis and PCAS (and by some also in trauma [6]) entitled disseminated intravascular coagulation (DIC) [7–10]. Acutely critically ill patients with coagulopathy have been reported to have three to four times higher mortality rates than their counterparts without coagulopathy, translating into a mortality rate of approximately 50%, which has remained virtually constant for decades [4, 7, 10].

In studies of trauma patients, increasing injury severity score (ISS) is associated with progressive hypocoagulability [11, 12]. This could be regarded as counterintuitive from an evolutionary perspective, as these patients are at high risk of exsanguination and, therefore, would need an intact or even improved hemostatic capacity of blood flow. We have proposed that the coagulopathy observed in these patients is a compensatory mechanism counterbalancing the shock-induced pro-thrombotic vascular endothelium in the microcirculation in order to secure sufficient organ perfusion in conditions with shock [12, 13]. Importantly, systemic endothelial injury seems pivotal for the development of organ failure and ensuing poor outcome [14, 15], pointing to a possible explanation of the association between coagulopathy and poor outcome in acute critical illness [8, 10, 16, 17].

The endothelium is one of the largest “organs” in the body, with a total weight of approximately 1 kg and a surface area of approximately 5000 m^2^ [18]. Endothelial cells form the innermost lining of all blood and lymphatic vessels and extend to all reaches of the vertebrate body. Far from being an inert layer of nucleated cellophane, the endothelium partakes in a wide array of physiological functions, including control of vasomotor tone, maintenance of blood fluidity, regulated transfer of water, nutrients and leukocytes across the vascular wall, innate and acquired immunity, angiogenesis and establishment of a unique dialogue between the underlying tissue and the flowing blood [18]. It is also recognized that the endothelium plays a critical role in a multitude of diseases, such as arteriosclerosis, malignancy and acute inflammatory diseases either as a primary determinant of pathophysiology or as a victim of collateral damage [19, 20].

Under normal conditions the endothelium is anticoagulated by a number of natural anticoagulant systems including the negatively charged luminal surface layer, the glycocalyx, which is rich in heparonoids and interacts with antithrombin [21]. Furthermore, tissue factor pathway inhibitor (TFPI) and the protein C/thrombomodulin system also contribute to endothelial anticoagulation along with endothelial release of tissue-type plasminogen activator (tPA) and urokinase-type plasminogen activator (uPA) that dissolves forming clots [22]. Hence, we propose that shedding, degradation and/or release of the glycocalyx and the natural anticoagulant and pro-fibrinolytic factors from the injured endothelium induces the profound hypocoagulability observed in acute critically ill patients with shock [12].

**Fig. 1:**
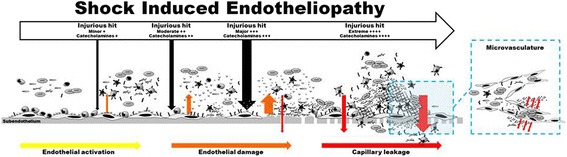
Shock-induced endotheliopathy (SHINE). Schematic illustration of the changes in the vascular compartment with increasing disease severity and increasing sympatho-adrenal activation (Original figure)

In trauma patients, TIC is present already at the scene of the accident in the most severely injured, shocked patients [23] indicating a potential contribution of the sympatho-adrenal system to this “early” coagulopathy. Cannon described in 1915 how the hormone adrenaline, released immediately upon severe stress, mobilizes an emergency response denoted the “fight or flight” response, and furthermore that the sympatho-adrenal activation “orchestrates changes in blood supply, sugar availability and the blood’s clotting capacity in a marshalling of resources keyed to the violent display of energy” [24]. We propose that the shock-induced sympatho-adrenal hyperactivation and ensuing excessive increase in circulating levels of catecholamines, not only activates but also directly inflicts systemic damage to the endothelium, including the microcirculation [25, 26]. Apart from the obvious increased risk of microvascular occlusion secondary to pro-thrombotic microcirculation in these patients, capillary leakage also significantly contributes to disease progression due to hypovolemia, edema, tissue hypoxia and exacerbated shock, resulting in a viscous circle with sustained sympatho-adrenal hyperactivation and release of large amounts of catecholamines, further compromising the microvasculature [27] (Fig. 1).

Here we describe and discuss the pathophysiology of shock-induced endotheliopathy (SHINE), a proposed new disease entity with unifying pathological change observed in acutely critically ill patients challenged by shock.

### Shock-induced endotheliopathy (SHINE)

We propose that shock, and its effect on the sympatho-adrenal system, the endothelium, including the glycocalyx and the hemostatic cells in the circulating blood results in phenotypic features that characterize the clinical condition of patients suffering acute critical illness, despite the different types of injurious “hit” they suffer [6, 9, 15, 27–30]. The catecholamine-induced damage to the endothelium is responsible for endothelial breakdown resulting in glycocalyx shedding, breakdown of tight junctions with capillary leakage and a pro-coagulant microvasculature that further reduces oxygen delivery due to increased tissue pressure and microvascular thrombosis creating a vicious circle that ultimately results in organ failure. The early genetic responses to severe trauma, burn injury and endotoxemia are similar [31], indicating that the response mounted by the body to various acute critical conditions accompanied by shock, is relatively homogenous and most likely evolutionarily adapted [12].

### Endotheliopathy of traumatic shock

We have investigated the degree of coagulopathy, sympatho-adrenal activation (plasma catecholamines) and endothelial injury (circulating biomarkers of endothelial cell (soluble thrombomodulin (sTM)) and glycocalyx (syndecan-1) damage) in three independent cohorts of severely injured patients (total number 579) [5, 16, 32–35]. Here we found strong and independent associations between high injury severity, high plasma adrenaline level, profound hypocoagulability and high circulating syndecan-1 and sTM levels. High plasma adrenaline was a strong and independent predictor of increased mortality [32] and hypocoagulability [36] and, importantly, despite comparable injury severity, trauma patients with the highest syndecan-1 levels (reflecting the highest degree of glycocalyx damage) had several-fold higher mortality [16, 33]. This emphasizes the pivotal importance of the state of the endothelium for outcome in these patients and also points towards a possible genetic predisposition of the endothelial response to shock. Furthermore, we found a significantly different sympatho-adrenal and endothelial response to the injurious “hit” in older vs. younger trauma patients, indicating that patient age also appears to significantly influence the response that is mounted, including the degree of endotheliopathy [37]. This is in accordance with the well-described association between higher age and progressive disruption and dysfunction of the endothelium, with the most profound endothelial disruption observed in smokers and patients with diabetes, hypertension or atherosclerosis [20, 38]. In addition to age, gender also significantly influences the endogenous trauma shock response [39] and both age and male gender are strong and independent predictors of multiple organ failure, an outcome closely linked to endotheliopathy, following severe trauma [40].

The critical importance of glycocalyx shedding in TIC was further illustrated by our finding that the most severely injured trauma patients displayed evidence of endogenous heparinization, as evaluated by whole blood thrombelastography (TEG) [35]. Endogenous heparinization is the result of the shedding of the glycocalyx, including heparan sulphate having the same functional effects as heparin on the hemostatic system. Also, damage to the endothelial cells induces release of thrombomodulin in its soluble form, which retains its anticoagulant effects also when circulating in the blood. Patients with evident endogenous heparinization displayed four-fold higher plasma syndecan-1 levels, strongly indicating that release of heparin-like constituents from the glycocalyx induced the endogenous heparinization. Patients with endogenous heparinization also had higher transfusion requirements, higher sTM levels and lower protein C levels compared to patients without endogenous heparinization. This emphasizes that the endotheliopathy included both extensive endothelial cell and glycocalyx damage [35, 41–44]. It should be noted, however, that these intriguing data are only observations and as such are hypothesis-generating, and currently there is no firm evidence available from RCTs to clarify whether endotheliopathy merely reflects greater disease severity, which in turn is known to relate to more organ dysfunction, or a severity-independent association with organ injury.

### Endotheliopathy of septic shock

Septic coagulopathy evidenced by DIC has for decades been associated with poor outcome [7, 8] and the accompanying endothelial dysfunction and injury are both hallmarks and drivers of the poor outcome [8, 29]. Based on the hypothesis that coagulopathy is a surrogate marker and a result of systemic endotheliopathy, we conducted a study investigating patients (n = 321) with varying degrees of infectious disease ranging from systemic inflammatory response syndrome (SIRS) without infection or with local infection, to sepsis, severe sepsis or septic shock [45]. Here we found that plasma syndecan-1 and sTM increased progressively and significantly across groups with increasing infectious severity and correlated significantly with organ failure as measured by the sequential organ failure assessment (SOFA) score in all groups. Furthermore, plasma levels of catecholamines, syndecan-1 and sTM were significantly higher in non-survivors compared to survivors and high levels of both catecholamines, syndecan-1 and sTM were all independent predictors of excess mortality, linking sympatho-adrenal hyperactivation and endothelial damage to outcome in patients with sepsis.

Patients with septic shock per definition receive vasopressor treatment, most often noradrenaline. Given this, it could be speculated whether the high therapeutic noradrenaline concentrations further promote endotheliopathy in these patients. We investigated this in a small study of patients (n = 67) of whom 21% received noradrenaline infusion at the time of blood sampling [46]. The study demonstrated that the levels of a broad range of biomarkers reflecting endothelial damage, including syndecan-1 and sTM, did not differ between patients with or without noradrenaline infusion, indicating that endotheliopathy in patients with septic shock was not further aggravated by catecholamine infusion [46].

Similarly, there was a strong association between endotheliopathy and organ failure in a large multicenter study of 1103 critically ill patients predominantly suffering from sepsis [47], demonstrating that patients with sepsis had higher plasma levels of syndecan-1 and sTM (more excessive endothelial damage) than non-infected patients. When stratifying the patients into quartiles based on sTM levels at study enrollment, mortality could be differentiated across all four quartiles during the entire follow-up period, with the highest mortality in the highest sTM quartiles, even after adjusting for other prognostic variables. Importantly, high syndecan-1 and sTM levels independently predicted liver and renal failure, respectively, and high sTM was further associated with increased risk of development of multiple organ failure. In sensitivity analysis, a composite endpoint of “circulatory failure or death” was created to overcome potential lead bias, as inotropic/vasopressor drugs are often removed from patients bound to die. After adjusting for confounders, both syndecan-1 and sTM study enrollment independently predicted the risk of “circulatory failure or death”, further pointing towards the central role of endotheliopathy for the pathophysiology related to outcome in patients with septic shock [47].

Finally, in a smaller cohort of 184 patients with severe sepsis or septic shock we found an independent association between high circulating syndecan-1 levels and coagulopathy evaluated by TEG, further linking endotheliopathy and coagulopathy also in sepsis [45]. Though it has been evident for decades that endothelial injury is a hallmark of sepsis [8, 27, 29], new data keep emerging that further reveal the pathophysiology of endothelial cell and glycocalyx damage in sepsis and its association with disease severity, including the applicability of biomarkers for outcome [48–51]. Similar to traumatic endotheliopathy, the findings described here are observational and, hence, no causality can be inferred.

### Endotheliopathy of cardiogenic shock and cardiac arrest

Cardiac arrest is the ultimate ischemia-reperfusion “hit” to the body. PCAS represents the systemic response to the global ischemia-reperfusion injury [15], which involves profound endothelial injury and ensuing microcirculatory dysfunction and failure secondary to capillary leakage, tissue/organ edema and hypoxia and increased blood cell adhesion to the activated/injured endothelium. The consequence of this global ischemia-reperfusion injury to the endothelium is a sepsis-like inflammatory response [9, 15, 30] that ultimately drives organ failure similarly to that observed in sepsis.

In 2007, Rehm and colleagues provided the first evidence in humans for shedding of the endothelial glycocalyx in conditions with ischemia-reperfusion [52]. In three groups of surgical patients (patients undergoing thoracic aortic surgery with deep hypothermic cardiac arrest, patients undergoing cardiac surgery on cardiopulmonary bypass and patients undergoing surgery for an abdominal aortic aneurysm) it was found that global and regional ischemia was followed by an increase in both syndecan-1 and heparan sulfate, two constituents of the endothelial glycocalyx [52], a finding confirmed by later studies [53].

Patients resuscitated from cardiac arrest frequently demonstrate profound hypocoagulability and hyperfibrinolysis of the flowing blood along with shedding of the glycocalyx [54, 55]. In a post-hoc analysis of 163 patients included at our center, Rigshospitalet, in The Targeted Temperature Management at 33 degrees versus 36 degrees after Cardiac Arrest (TTM) trial [56], we found that catecholamines correlated strongly with syndecan-1 and sTM plasma levels i.e. biomarkers reflecting endothelial glycocalyx and cell damage [57]. Overall 180-day mortality was 35% and both plasma adrenaline and sTM levels were the strongest, and independent, predictors of mortality [57]. This finding is in line with our previous study of 678 patients with acute ST-elevation myocardial infarction (STEMI), demonstrating that admission levels of plasma adrenaline, syndecan-1 and sTM were highly correlated with the highest levels of adrenaline and syndecan-1 in patients with cardiogenic shock [38]. Furthermore, STEMI patients admitted to ICU displayed the highest syndecan-1 plasma levels and high levels of adrenaline, syndecan-1 and sTM were strong predictors of poor outcome, including heart failure and mortality [38].

Together these findings indicate that sympatho-adrenal hyperactivation and endothelial damage are inter-correlated and strong predictors of mortality in conditions with cardiogenic shock [38, 57], and furthermore that myocardial infarction alone appears also to inflict significant systemic endothelial damage, possibly driven in part by the parallel increase in circulating catecholamines, albeit evidence from prospective randomized trials are lacking [38]. The finding, however, is in alignment with previous studies reporting high circulating levels of glycocalyx components (syndecan-1, heparan sulphate) in patients with cardiogenic shock, with high levels being strong predictors of excess mortality [58].

## Discussion

In the observational data presented here from more than 3000 patients with different types of acute critical illness including different types of shock, high circulating catecholamine levels are independently associated with endotheliopathy and are predictive of poor outcome (both short-term and long-term mortality) and, furthermore, that this shock-induced endotheliopathy is statistically linked to the development of organ failure and death. Given that shock and endothelial disruption and damage coincide in patients with the most severe form of acute critical illness, a mechanistic link is suggested between sympatho-adrenal hyperactivation and the endothelial phenotype, and that this shock-induced endotheliopathy (SHINE), may be a unifying pathophysiologic mechanism, linked to outcome, albeit this awaits further confirmation [12, 28].

Recently, a link between sympatho-adrenal hyper-activation and endothelial damage was suggested in an animal model of trauma shock demonstrating that both chemical sympathectomy and treatment with β-blockade attenuate endothelial glycocalyx and endothelial cell damage in rats with acute traumatic coagulopathy [59]. This may provide an explanation for the limited success of many large RCTs conducted in acutely critically ill patients in the past decades [60]. Among patients with severe sepsis/septic shock alone, more than 30,000 patients have been enrolled in clinical trials to test anti-coagulant, anti-inflammatory, anti-endotoxin and immune-modulating agents [60, 61]. Yet, not a single agent has convincingly proven to be consistently efficacious and there are still no new drugs on the market with the indication of sepsis, despite tremendous effort worldwide. Similarly, in patients suffering from out of hospital cardiac arrest (OHCA), two small RCTs (77 and 136 patients, respectively) conducted in 2002 reported improved survival in those receiving therapeutic hypothermia targeted at approximately 33 °C [62, 63]. However, in a large RCT including 939 patients randomized to temperatures of 33 °C or 36 °C, there was difference between groups in mortality [56], and a recent meta-analysis of RCTs reported no benefit of mild therapeutic hypothermia on neurologic outcome or mortality in patients who had OHCA [64].

In trauma, mortality has been reduced substantially in the past 10–15 years as a result of the introduction of damage control surgery and hemostatic resuscitation [65–67]. A recent multicenter RCT in trauma patients with severe hemorrhage demonstrated a significant reduction in early mortality caused by exsanguination, with more aggressive administration of plasma and platelets [68]. Similarly, a recent RCT was prematurely halted due to a significantly increased survival of patients who were resuscitated aggressively based on whole blood TEG compared to conventional coagulation assays [69]. Unfortunately, the excess mortality in patients with TIC has remained unchanged by these improvements, highlighting a therapeutic failure here as well.

Given the potential unifying pathologic condition of SHINE across patients with different types of acute critical illness, it could be speculated whether interventions targeting the endothelium and/or the sympatho-adrenal system could be of value here. By 1978, β-blocker therapy had already been reported to have beneficial effects on MI [70] and in a later meta-analysis of RCTs investigating the use of early intravenous beta-blockers in patients with acute coronary syndrome there were significant reductions in the risk of short-term cardiovascular events, including reduction in all-cause mortality [71].

The beneficial effects of β-blocker therapy in these patients have historically been envisioned to be related to reductions in the incidence of arrhythmia and improved cardiac myocyte function. However, we speculate that blockade of the effects of the catecholamine surge on the endothelium, and hereby reduced systemic endotheliopathy, may also have contributed to the improved outcome and this should be investigated further. In a recent small RCT of patients with septic shock and heart rate above 95 beats per minute, 77 patients were randomized to either short-acting β-blocker therapy with Esmolol to maintain heart rate between 80 and 94 beats per minute during their ICU stay or to placebo [72]. Patients receiving β-blocker therapy had lower 28-day mortality compared to the control group (49% vs. 81%, adjusted hazard ratio of 0.39).

Taken together these results may indicate that sympathoadrenal hyper-activation may be hazardous for acute critically ill patients and according to our proposed hypothesis, use of β-blocker therapy in these previous trials may have prevented or reduced the catecholamine-induced endotheliopathy, which translated into improved survival in patients suffering from cardiac disease including cardiac arrest, trauma and sepsis. Adequately powered RCTs are necessary to confirm or reject this hypothesis.

## Conclusion

Shock-induced endotheliopathy (SHINE) is observed in acute critical illness and may reflect a potential unifying pathophysiologic mechanism linked to poor outcome. Sympatho-adrenal hyperactivation appears to be a pivotal driver of this condition.

## Abbreviations

DIC: Disseminated intravascular coagulation
MI: Myocardial infarction
OHCA: Out-of-hospital cardiac arrest
PCAS: Post cardiac arrest syndrome
RCT: Randomized controlled trial
SHINE: Shock-induced endotheliopathy
SIRS: Systemic inflammatory response syndrome
SOFA: Sequential organ failure assessment
sTM: soluble Thrombomodulin
TEG: Thrombelastography
TFPI: Tissue actor pathway inhibitor
TIC: Trauma-induced coagulopathy
TTM: Targeted temperature management
