# Erratum to: Shock induced endotheliopathy (SHINE) in acute critical illness - a unifying pathophysiologic mechanism

**DOI:** 10.1186/s13054-017-1756-4

**Published:** 2017-07-13

**Authors:** Pär Ingemar Johansson, Jakob Stensballe, Sisse Rye Ostrowski

**Affiliations:** 1 Capital Region Blood Bank, Rigshospitalet Section for Transfusion Medicine, Rigshospitalet, Copenhagen University Hospital, Blegdamsvej, 9DK-2100 Copenhagen, Denmark; 2 Department of Surgery, University of Texas Health Medical School, Houston, TX USA; 3 Centre for Systems Biology, The School of Engineering and Natural Sciences, University of Iceland, Reykjavik, Iceland; 4 Department of Anesthesia, Centre of Head and Orthopedics, Rigshospitalet, Copenhagen University Hospital, Copenhagen, Denmark

## Erratum

Unfortunately this article [1] was published with an error. The first and last author names are presented incorrectly. The first author name should be Pär Ingemar Johansson, or alternatively Johansson PI. The last author name should be Sisse Rye Ostrowski, or alternatively Ostrowski SR.
